# A proposal for a new concept in psychiatric phenomenology: The “Yume‐Utsutsu” (dreamy or half‐asleep) phenomenon in patients with dementia with Lewy bodies

**DOI:** 10.1002/pcn5.50

**Published:** 2022-10-18

**Authors:** Katsuyuki Ukai

**Affiliations:** ^1^ Department of Psychogeriatrics Kamiiida Daiichi General Hospital Nagoya Japan; ^2^ Department of Psychiatry Nagoya University Graduate School of Medicine Nagoya Japan

**Keywords:** automatism, dementia with Lewy bodies, psychopathology, rapid eye movement sleep behavior disorder, Yume‐Utsutsu phenomenon

## Abstract

**Background:**

Patients with dementia with Lewy bodies (DLB) present with a variety of physical and mental symptoms, including visual hallucinations, delusions, depression, rapid eye sleep movement behavior disorder, and olfactory dysfunction. This study describes another interesting psychiatric symptom, which seems to be characteristic of DLB, in many patients who visited the author's psychogeriatrics department for neurocognitive disorders or psychogeriatric diseases.

**Methods:**

The clinical courses of seven representative cases of DLB, each of which included episodes of this interesting psychiatric symptom, are described to clarify the commonalities they shared.

**Results:**

After examining the characteristic symptoms of these cases, a novel term was proposed for a new concept in psychiatric phenomenology, the “Yume‐Utsutsu” (dreamy or half‐asleep) phenomenon. “Yume‐Utsutsu” is a Japanese word that means a mental state in which dreams are indistinguishable from reality. This phenomenon is different from visual hallucinations, fluctuating cognition, rapid eye sleep movement behavior disorder, delirium, and epilepsy‐related automatism.

**Conclusions:**

The “Yume‐Utsutsu” (dreamy or half‐asleep) phenomenon proposed as a novel psychiatric concept in this article is useful for diagnosing and clarifying the psychopathology of DLB.

## INTRODUCTION

Patients with dementia with Lewy bodies (DLB) present with a variety of physical, psychological, and behavioral symptoms. For example, the physical symptoms of DLB include parkinsonism, olfactory dysfunction, syncope, and pain, the psychological symptoms include hallucinations, delusions, misidentification, and depression, and the behavioral symptoms include rapid eye movement sleep behavior disorder (RBD), suicide attempts, and abnormalities based on delusions and misidentification. Most of these symptoms have been adopted in the revised criteria for the clinical diagnosis of DLB.^1^

The author has often experienced cases involving an interesting psychiatric symptom that seems to differ from any of the previously described symptoms of DLB. This paper proposes this symptom as a new psychiatric phenomenon in psychiatric phenomenology (Phänomenologie des Geistes, Psychopathology).

## METHODS

The author has experienced many DLB cases in which the abovementioned symptom was observed. Here, the clinical courses of seven representative cases of DLB, each of which included episodes of the interesting psychiatric symptom mentioned above, are described to clarify the commonalities they shared.

Informed consent to publish the patients' clinical information was obtained from the patients and their families. Minor modifications to the data, which did not interfere with the study's findings, were made to preserve the patients' anonymity.

## RESULTS (CASE PRESENTATION)

### Case 1

The patient was a male in his late 70s. His medical history did not include any relevant conditions, except for recurrent nocturnal dream‐enactment behavior (i.e., RBD), which had first developed in 2016. About 2 months later, he started to experience visual hallucinations (VH), in which he saw some strangers. In 2017, he visited a memory hospital and underwent cranial magnetic resonance imaging (MRI) and 123I‐meta‐iodobenzylguanidine (MIBG) myocardial scintigraphy. Under a diagnosis of DLB, donepezil therapy was started at a dose of 5 mg/day.

Since his symptoms did not improve markedly, he and his family consulted the author's clinic in 2018. His Mini‐Mental State Examination (MMSE) score was 22. The donepezil dose was increased to 10 mg/day and his VH completely disappeared. However, both he and his family complained that the patient did not know if he was in a dream or in reality when he woke up (this even applied to waking from short naps). Some examples of this are provided: (1) He was talking with a stranger in his dream and then woke up. However, he continued to talk although nobody was there, and then he began to look for the stranger in front of his family for a while. (2) One night, he suddenly entered his son's bedroom and said, “I have to go to my office” (he had retired many years before), “I received a telephone call.” Regarding these behaviors, he explained in my examination room, “Dreams continue even if I wake up. I cannot recognize whether I am in a dream or reality even after I wake up. I feel like I am half asleep.”

### Case 2

The patient was a male in his early 70s with no relevant medical history. His wife had died about 10 years ago and he lived with his daughter's family. He developed RBD in about 1993 and VH in 2017. He and his daughter visited a memory hospital, he was diagnosed with DLB, and donepezil treatment was started. After the initiation of donepezil treatment, his VH became mild, but he and his daughter consulted the author's department in 2018. His MMSE and Montreal Cognitive Assessment (MoCA‐J) scores were 28 and 24, respectively.

In 2020, his VH became severe, and tactile hallucinations also appeared, including a feeling of his hair being touched by a stranger, a sticky feeling when touching a bird like a crane, and a feeling of grabbing crumpled hair on the floor.^2^ About 6 months after that, he complained in the author's examination room that dreams and reality were becoming mixed up. For example: (1) He said that one day he talked with his wife (who had died) in a dream and then woke up in reality. Even after that, he continued talking with her. After a while, he began to wonder if it might be an illusion. (2) One night, his daughter was suddenly awakened in her bedroom by the patient, and he said, “I have to prepare for the dinner party. Let's go early. If we are late, it is no good.”

### Case 3

The patient was a male in his early 80s with no relevant medical history. He developed VH of strangers in 2015. He visited a memory hospital and underwent cranial MRI, single‐photon emission computed tomography (SPECT), and MIBG myocardial scintigraphy. His MMSE score was 22, and he was prescribed donepezil under a diagnosis of DLB. After about 3 months, he and his family consulted the author's clinic. His MMSE score had improved to 28 and the VH had also disappeared. The donepezil therapy was continued.

In 2019, the patient and his family complained that the patient had begun to fall asleep and have difficulty distinguishing between dreams and reality when he woke up. For example: (1) In a dream, he was consulted by his subordinate on a business matter (he had retired many years before). He woke up and then woke up his wife and said to her, “What shall I do, that problem is too difficult for me”. After a while, he remembered that he had been retired for many years. (2) One night, he woke his wife up and said, “My friend came here and asked to stay the night. Put the duvet out for him.” After a while, he could see that it was a dream. (3) Another night, he woke up and said to his wife, “I have to go to my factory. The machine has been left running!” His wife spent more than 5 min making him realize that it was a dream. His wife complained that similar episodes had happened several times.

### Case 4

The patient was a female in her late 70s with a medical history of hypertension. She developed RBD in about 2006 and VH in February 2016. She visited a hospital with her family and was suspected to have DLB, and she was referred to the author's department in July 2016. Mild parkinsonism and olfactory disturbance were observed. Her MMSE score under treatment with 5 mg/day donepezil was 25. Cranial MRI and electroencephalography (EEG) revealed no abnormal findings. Her main VH was of unknown children, who were often crying or singing and would sometimes pull at her clothes (auditory and tactile hallucinations were also observed). After the donepezil dose was increased to 10 mg/day, the VH and other hallucinations disappeared.

In 2020, the VH relapsed, and the patient and her family complained that she had begun to fall asleep and have difficulty distinguishing between dreams and reality after she woke up. For example, she got up one night and began to change her clothes. Her husband asked her, “What are you doing here at this time of night?” The patient answered, “I have to go to a meeting now.” The author confirmed in his examination room that she had dreamt that she had been called to a meeting. Several similar episodes had also been observed by her husband.

### Case 5

The patient was a male in his late 70s with a medical history of pectoralis angina. He developed severe RBD in 2013, and his family brought him to the author's clinic, as they worried about his symptoms, in 2014. Mild parkinsonism and olfactory disturbance were noted, but no clear VH were reported. His MMSE and MoCA‐J scores were 25 and 20, respectively. Cranial MRI and EEG revealed no significant abnormal findings. MIBG myocardial scintigraphy revealed disturbed MIBG uptake, and dopamine transporter (DAT)‐SPECT demonstrated reduced uptake in the basal ganglia. He was diagnosed with probable DLB and donepezil therapy was administered. After this treatment, his MMSE and MoCA‐J scores improved to 30 and 26, respectively.

In 2020, VH of an unknown sitting woman began. Several months after that, abnormal dream‐related behaviors also developed. For example: (1) One night, he woke up and began to look for something, shouting “What I had suddenly disappeared!” After a while, he said to his wife “I was looking for my business documents in a dream. I feel like a dream and reality got mixed up.” (2) Another night he woke up and said to his wife, “Come on, let's go. We have to go there.” She said to the patient, “You are in bed now. It is your dream.” He was confused for a while and said to her, “Oh right, a dream.”

### Case 6

The patient was a male in his late 60s with a history of recurrent RBD, which had started several years earlier. His VH, which included people coming to his home, water being spilt on the floor, and his desk going on fire, had begun in 2007. As his VH became more severe, he consulted the author's clinic with his family in 2009. Mild parkinsonism and a severely seborrheic face were noted. Cranial MRI and EEG did not show any abnormalities. His MMSE score was 27, and his Alzheimer's Disease Assessment Scale‐cognitive component (ADAS J‐cog) score was 15.3. Based on the findings of clinical examinations, cerebral blood flow SPECT, and MIBG myocardial scintigraphy, he was diagnosed with mild neurocognitive disorder with Lewy bodies. The administration of donepezil resulted in the complete disappearance of his VH. His MMSE and ADAS J‐cog scores improved markedly to 30 and 5.0, respectively.

In about 2008, about 1year before they first visited the clinic, his wife and daughter mentioned that they had often observed him wandering the house at night. One night he had left his house and a few hours later he came back home with policemen. At that time, he said to his family, “I found myself in a place I do not know. The policemen helped and brought me here. I felt like I was taking a walk in my dream.”

### Case 7

The patient was a female in her late 60s with a medical history of hypertension and hyperlipidemia. In 2012, recurrent RBD and olfactory disturbance began. She and her family consulted the author's clinic in 2015 because memory impairment appeared. On examination, mild lead‐pipe rigidity of the bilateral upper extremities and impairment of the postural reflexes were observed. Her MMSE and MoCA‐J scores were 30 and 24, respectively. MRI and EEG revealed no abnormal findings. However, she fell and fractured her femur, and underwent surgery at another hospital. This led to a 3‐year interruption to her visits to the author's department.

In 2018, she and her family visited the author's department again. She complained of feeling someone was near her. Her MMSE and MoCA‐J scores were 28 and 22, respectively. No abnormal findings were found on MRI or EEG. Although she rejected further examinations, such as DAT‐SPECT and MIGB myocardial scintigraphy, she was diagnosed with mild neurocognitive disorder with Lewy bodies. During a subsequent examination, she complained that at night she found herself in the middle of the stairs of her house. She could not understand why she was there, but she felt like she had been sleep‐walking. Her third EEG also showed no abnormal findings.

## DISCUSSION

### A proposal for a new concept in psychiatric phenomenology

Some clinicians, such as Kenji Kosaka and Manabu Ikeda, who have examined many patients with DLB, are already aware of the symptoms described above, but to the best of the author's knowledge, this phenomenon has not been specifically described in the literature. The psychiatric phenomenon being proposed here is that after waking up from sleep DLB patients perceive that the events in their dreams are still continuing in reality, and in the real world they continue to behave as they did in their dreams. The patients themselves often remember these illusions well afterwards and complain at their next clinic visit that they could not tell whether they were in a dream or reality.

The author regards this psychiatric phenomenon as being different from RBD, delirium, and epilepsy‐related automatism and calls it the “Yume‐Utsutsu” (dreamy or half‐asleep) phenomenon. “Yume‐Utsutsu” is a Japanese word that refers to a mental state in which it is difficult to distinguish between dreams and reality.

### Characteristics and commonalities of cases involving the “Yume‐Utsutsu” phenomenon

1) Of the presented cases, five (cases 1–5) involved DLB (major neurocognitive disorder with Lewy bodies) and two (cases 6 and 7) involved mild neurocognitive disorder with Lewy bodies.
2) All of the patients that experienced this phenomenon also demonstrated RBD.
3) All of the patients were able to specifically explain their experiences of this phenomenon in the examination room at least several weeks after the episodes. They remembered the episodes well.
4) The patients, especially those with DLB, complained that they did not know whether they were in a dream or reality, even after they woke up, and that they felt that their dreams and reality had become mixed up. The term “half‐asleep” is a term that one patient expressed.
5) It seems that the “Yume‐Utsutsu” phenomenon tended to occur in patients with DLB when their VH and/or cognitive functions became worse.
6) There may be differences between the symptoms experienced by DLB patients and those experienced by patients with mild neurocognitive disorder with Lewy bodies. The patients with mild neurocognitive disorder with Lewy bodies had been walking around half‐asleep and then seemed to come back to reality. Both of these patients explained that they could not understand why they were in the locations they were when they came around. Although they complained that they felt like they were taking a walk in their dreams, it is not certain that this was a behavior that followed from their dreams. Further research is required regarding this point.

### Differences between the “Yume‐Utsutsu” phenomenon and RBD

RBD is an abnormal behavior seen during sleep.^3^ Patients with RBD show sleep‐talking and body movements that are consistent with their dream contents. Their companions can usually confirm this by waking the patient and asking them about the contents of their dreams. In addition, the patients themselves can recognize their actions as being due to events in their dreams. For example: (1) When an RBD patient is fighting a robber in their dream by loudly threatening, punching, and kicking the robber, their companion can observe them shouting and flailing their arms and legs. (2) When an RBD patient is speaking in an office meeting in a dream, in the real world their partner can see that they are talking in their sleep as if they are talking with someone else.

On the other hand, the “Yume‐Utsutsu” phenomenon is an abnormal behavior seen immediately after waking up. Patients that experience this phenomenon behave as if they are still in their dream world, despite having woken up, as was described in the cases above.

RBD can be accurately diagnosed by confirming the presence of rapid eye movement sleep without atonia (RWA) with polysomnography. However, polysomnography is not so useful for distinguishing RBD from the “Yume‐Utsutsu” phenomenon because most DLB patients also have RBD. It is usual for DLB patients to be confirmed to have RWA by polysomnography.^3^

### Differences between the “Yume‐Utsutsu” phenomenon and delirium

Delirium is a temporary condition caused by brain dysfunction that involves mild to moderate impairment of consciousness and is often associated with VH. Many patients with DLB also experience VH, but their consciousness is not impaired if it is not complicated with delirium. When listening to DLB patients' clinical stories, clinicians should take care to distinguish whether the patient is talking about a dream, hallucination, or delirium. In the case of a dream or hallucination, the patient would usually remember it well. However, in the case of delirium the patient would not remember it well because their consciousness would have been impaired. Hence, the patient would not be able to explain their actions adequately.

Patients that experience the “Yume‐Utsutsu” phenomenon do not have impaired consciousness and are able to explain the reasons for their behaviors later in the examination room. Therefore, one of the core features of the clinical diagnostic criteria for DLB, “fluctuating cognition with pronounced variation in attention and alertness”, can be considered as not associated with the “Yume‐Utsutsu” phenomenon.^1^

### Differences between the “Yume‐Utsutsu” phenomenon and automatism

Elderly people sometimes exhibit automatism during sleep because they are often complicated with epilepsy attacks, especially focal impaired awareness seizures (FIAS).^4^ A few previous studies have suggested that DLB patients often develop FIAS.^5^, ^6^ Automatism associated with FIAS is sometimes difficult to distinguish from RBD and delirium at night, even for clinicians. However, because patients with automatism and/or delirium have impaired consciousness they are not able to remember their behaviors at night. On the other hand, patients with RBD or the “Yume‐Utsutsu” phenomenon can remember and explain the reasons for their behaviors later (see Section 4.3 for more information on differentiating between the “Yume‐Utsutsu” phenomenon and RBD).

When it is difficult to distinguish between this phenomenon and automatism during sleep, diagnostic anti‐epileptic drug (AED) therapy and EEG examinations may be useful. In general, AEDs work well for FIAS that arises in the elderly.

### The frequency and timing of the development of this phenomenon

It would have been useful as one of the original purposes of this study to examine the frequency and timing of the development of the “Yume‐Utsutsu” phenomenon. Unfortunately, this was not done because time constraints during clinical consultations did not allow for questioning of all patients with DLB about this phenomenon, especially during the COVID‐19 pandemic.

However, based on clinical impressions, the author considers that about a quarter to half of patients with DLB complain of this phenomenon during their clinical courses, and it seems that this phenomenon tends to occur in patients with DLB when their VH and/or cognitive functions become worse. It is hoped that this phenomenon can be brought to the attention of many clinicians and that the abovementioned problems will be resolved in the near future. Recognizing this phenomenon will help clinicians to diagnose and clarify the psychopathology of DLB.

### Why has this common phenomenon not been reported before?

The author first recognized this phenomenon about 5 years ago, but the memory clinic had been started in 2005, this means that this phenomenon had been overlooked for at least 10 years. At first, the phenomenon was considered to be an atypical type of RBD, but this was a misconception because RBD should only occur when patients are sleeping, that is, dreaming.

Patients rarely complain of this phenomenon voluntarily, and most clinicians are too busy to ask patients about their symptoms in detail (at least recently in Japan).

The author was a pupil of Kenji Kosaka, who discovered and established the entity of DLB, and one of the core members of the Japan DLB Research Association established in 2007 and the DLB Family Association in Japan established in 2008, both of which were organized by Kosaka. Therefore, many DLB patients and their relatives have visited the author's clinic since 2008, and the author has examined many patients with DLB. This situation and teaching from Kosaka are the main reasons why the author noticed this phenomenon.

### Limitations of this study

(1) The frequency and timing of the development of the “Yume‐Utsutsu” phenomenon should be clarified in the near future.

(2) It is important to clarify whether the symptoms seen in cases 6 and 7 differ from the typical “Yume‐Utsutsu” phenomenon observed in cases 1–5.

(3) If the symptoms seen in cases 6 and 7 are different from the “Yume‐Utsutsu” phenomenon, the mechanisms responsible for these symptoms should be examined, for example are they epilepsy‐related symptoms or a new type of sleep‐wake disorder with DLB?

## CONCLUSIONS

1) A new conceptual term is proposed, the “Yume‐Utsutsu” (dreamy or half‐asleep) phenomenon, which refers to a mental condition in which DLB patients become unable to determine whether they are in a dream or reality, even after waking up. To the best of the author's knowledge, this condition has not yet been specifically described in the literature.
2) This condition can be distinguished from RBD by checking whether the patient behaves in the same way after waking up, that is, as if they are still in their dream, or is able to recognize that it was a dream.
3) This condition can be distinguished from delirium by checking whether the patient's consciousness was normal or impaired at the time, as well as by asking whether the patient remembers the episode at their next examination.
4) This condition can also be distinguished from FIAS‐associated automatism during sleep by checking whether the patient's consciousness was normal or impaired at the time of the episode, as well as by asking whether or not the patient remembers the episode at their next visit.

## CLINICAL TRIAL REGISTRATION

N/A.

## AUTHOR CONTRIBUTION

Katsuyuki Ukai is the only author of this article.

## CONFLICT OF INTEREST

The author declares no conflict of interest.

## ETHICS APPROVAL STATEMENT

The study was approved by the Ethics Committee of Kamiiida Daiichi General Hospital (Nagoya, Japan).

## PATIENT CONSENT STATEMENT

Informed consent to publish the patients' clinical information was obtained from the patients and their families.

## Data Availability

On reasonable request, derived data supporting the findings of this study are available from the corresponding author after approval from the Ethical Committee.

## References

[1] McKeith IG , Boeve BF , Dickson DW , Halliday G , Taylor JP , Weintraub D , et al. Diagnosis and management of dementia with Lewy bodies. Fourth consensus report of the DLB consortium. Neurology. 2017;89:88–100. 28592453 10.1212/WNL.0000000000004058 PMC5496518

[2] Ukai K . Tactile hallucinations in dementia with Lewy bodies. Psychogeriatrics. 2019;19:435–9. 30734409 10.1111/psyg.12407

[3] Fujishiro H , Ota K , Yamagata M , Ito T , Hieda S , Suga H , et al. Early diagnosis of prodromal dementia with Lewy bodies using clinical history of probable REM sleep behavior disorder and cardiac ^123^I‐MIBG scintigraphy in memory clinics. Psychogeriatrics. 2021;21:288–95. 33565213 10.1111/psyg.12662

[4] Fisher RS , Acevedo C , Arzimanoglou A , Bogacz A , Cross JH , Elger CE , et al. A practical clinical definition of epilepsy. Epilepsia. 2014;55:475–82. 24730690 10.1111/epi.12550

[5] Ukai K , Fujishiro H , Watanabe M , Kosaka K , Ozaki N . Similarity of symptoms between transient epileptic amnesia and Lewy body disease. Psychogeriatrics. 2017;17:120–5. 27356810 10.1111/psyg.12199

[6] Ukai K , Ito M , Watanabe M . Transient epileptic amnesia accompanied by prodromal symptoms of dementia with Lewy bodies: the second case report in the literature. Psychogeriatrics. 2019;19:622–3. 30773739 10.1111/psyg.12434

